# Applying Proteogenomics to Characterize Molecular Features Associated With a High‐Risk HOXB13 Gene Variant in Prostate Cancer Tumors

**DOI:** 10.1002/mco2.71078

**Published:** 2026-10-21

**Authors:** Kelsie Raspin, Zainab Noor, Adel T. Aref, James R. Marthick, Chol‐hee Jung, Shaun Donovan, Steve Williams, Peter G. Hains, Phillip J. Robinson, Rosemary L. Balleine, Qing Zhong, Roger R. Reddel, Liesel M. FitzGerald, Joanne L. Dickinson

**Affiliations:** ^1^ Menzies Institute for Medical Research University of Tasmania Hobart Tasmania Australia; ^2^ ProCan® Children's Medical Research Institute Faculty of Medicine and Health The University of Sydney Westmead New South Wales Australia; ^3^ Melbourne Bioinformatics The University of Melbourne Parkville Victoria Australia; ^4^ Sonic Healthcare Hobart Tasmania Australia

**Keywords:** gene signature, HOXB13, prostate cancer, prostate tumors, proteogenomic, rare variants

## Abstract

The rare HOXB13 G84E variant is one of few established high‐risk prostate cancer (PrCa) variants, yet the underlying molecular mechanisms by which it drives tumor development remain unknown. Prostate tumors from six heterozygous G84E variant carriers and 18 non‐carriers (wild‐type) were examined using the Human AmpliSeq Transcriptome Gene Expression Panel, and proteomes were generated by mass spectrometry (SWATH). We identified a 799‐gene expression profile and a 75‐protein expression profile in G84E‐positive tumors when compared with wild‐type tumors. Cluster analysis revealed a top‐ranked 23 protein profile, which mapped to lipogenesis pathways. Integration of transcriptome and proteome data revealed overlapping differential expression of three genes (*NCLN*, *NFIB*, and *VASN*), two of which have an established link to lipid metabolic pathways. Examination of publicly available transcriptome data from 493 primary PrCa tumors from the The Cancer Genome Atlas (TCGA)   tumors revealed 52 tumors with a similar differential expression profile for these three genes. Herein, an innovative proteogenomic approach was used to map molecular changes unique to HOXB13 perturbation in prostate tumors, revealing upregulation of genes in the steroid metabolic, lipid biosynthesis, and fatty acid metabolic pathways.

## Graphical abstract

Prostate tumors from six heterozygous G84E variant carriers (G84E positive) and 18 non‐carriers (wild‐type) were examined using the Human AmpliSeq Transcriptome Gene Expression Panel, and proteomes were generated by mass spectrometry (SWATH). We identified a 799‐gene expression profile and a 75‐protein expression profile in G84E‐positive tumors when compared with wild‐type tumors. Integration of transcriptome and proteome data revealed overlapping differential expression of three genes (*NCLN, NFIB*, and *VASN*), two of which have an established link to lipid metabolic pathways.

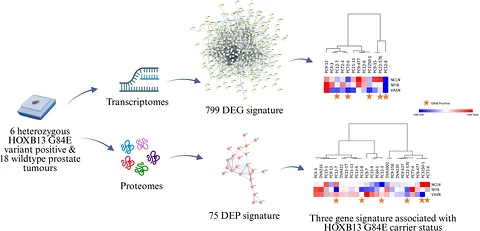

## 1 Introduction

PrCa has the highest heritability of any common cancer [1]; it is also the most frequently diagnosed non‐cutaneous malignancy in 118 countries [2]. We know that rare gene variants significantly contribute to risk; however, aside from the established rare variants in DNA damage repair genes (*BRCA1*, *BRCA2*, *ATM*), the rare G84E variant in *HOXB13* (rs138213197; chr17:48,728,343 C>T) remains the most frequently observed and most strongly associated PrCa risk variant. Originally identified in a familial PrCa cohort [3], this variant is associated with a 3–16‐fold increase in PrCa risk and has been reported in multiple studies, including our Tasmanian resource (OR = 6.59; *p* = 4.2 × 10^−5^) [4]. The G84E variant is associated with early‐onset familial disease [5, 6, 7, 8, 9, 10, 11]; however, there is no established association between carrier status and clinicopathological characteristics [3, 8, 12, 13], including Gleason score, a grading system used to assess PrCa aggressiveness—with a higher score indicating more aggressive disease.

HOXB13 is a transcription factor that is important for normal prostate development [14, 15] and is the only *homeobox* gene that maintains expression into adulthood [3, 14, 16, 17]. It has been postulated that HOXB13 can act as both an oncogene and tumor suppressor during carcinogenesis, and its role is dependent on the cell environment. HOXB13 plays a crucial role in the regulation of androgen receptor (AR) activity, a major regulator of the normal prostate and PrCa [18]. The G84E variant is located within the MEIS binding domain of HOXB13, which interacts with the histone deacetylase, HDAC3. Recently, Lu et al. identified HDAC3 as a key cofactor of HOXB13 and showed that together they reprogramme the epigenome and suppress the lipogenesis pathway in an in vitro model [19]. However, cell culture studies have indicated that the interaction between HOXB13 and MEIS1 is not impacted by the G84E variant [20]. Thus, the exact role of the variant remains unclear and, furthermore, it is yet to be determined whether G84E‐positive or indeed HOXB13 dysregulated prostate tumors represent a distinct molecular subtype of PrCa. Here, we aimed to determine whether prostate tumors harboring the heterozygous HOXB13 G84E rare variant have distinct proteome and transcriptome signatures.

There is growing evidence that other germline genetic variants, including rare variants, drive specific tumor transcriptome and proteome signatures (reviewed in Sadeesh et al. [21]). Defining “omic” signatures can stratify patients, reveal molecular drivers of disease, inform response to tailored therapies, and aid in the development of new therapeutics [22, 23]. An integrated multi‐omic approach has recently identified an 18‐protein signature, which is prognostic for risk of biochemical recurrence in intermediate‐risk PrCa patients [24]. Moreover, targeting protein function represents a successful approach for cancer therapeutics [25]. Therefore, a greater understanding of how the G84E variant impacts proteomic features may yield insights that will inform development of novel therapies. We hypothesized that a proteogenomic approach could identify key signatures and pathways associated with the G84E variant, potentially highlighting future areas of research focusing on novel therapeutic targets in those with HOXB13 dysregulated PrCa.

## 2 Results

### 2.1 Gene Expression Analysis of HOXB13 G84E Variant‐Positive and Wildtype Prostate Tumors

Array‐based transcriptome assays were performed for 12 prostate tumors from the Tasmanian studies, including five heterozygous HOXB13 G84E‐positive and seven wild‐type tumors. Table 1 describes the clinical characteristics of these tumors and Figure S1 shows that of the 17,128 gene transcripts that passed quality control (QC), ∼13,000 were expressed in >90% of samples.

**TABLE 1:** Clinical characteristics of prostate tumor samples available for this study.

Sample ID	Age at diagnosis	G84E tumor genotype	Tissue source	Tumor grade^a^	Gleason score^b^	% of Viable cancer	Malignant tissue available for transcriptome assays^c^
G84E non‐carriers							
DVA216	64	CC	RP	—	5 (3+2)	20–50	
DVA220	63	CC	RP	—	6 (3+3)	<20	
DVA422	61	CC	RP	—	7 (3+4)	20–50	
DVA1002	61	CC	RP	—	6 (3+3)	<20	
PC4‐3^*^	80	CC	TURP	M/PD	7 (4+3)	50–80	Y
PC9‐7	71	CC	TURP	—	9 (5+4)	50–80	
PC9‐12	66	CC	RP	—	6 (3+3)	<20	Y
PC9‐15	64	CC	TURP	—	9 (4+5)	80^^	Y
PC9‐158	63	CC	RP	—	6 (3+3)	<20	
PC9‐477	55	CC	RP	—	6 (3+3)	0^	Y
PC9‐627	65	CC	RP	—	7 (4+3)	20–50	
PC11‐11^*^	85	CC	TURP	—	7 (3+4)	20–50	Y
PC12‐1^*^	63	CC	RP	MD	6 (3+3)	<20	
PC12‐6^*^	80	CC	TURP	PD	7 (3+4)	<20	Y
PC12‐9^*^	68	CC	TURP	—	6 (3+3)	<20	
PC22‐17	56	CC	RP	—	6 (3+3)	<20	
PC60‐1^*^	58	CC	TURP	WD	6 (3+3)	<20	
PC72‐4^*^	70	CC	TURP	PD	9 (4+5)	>80^#^	Y
G84E variant carriers							
PC12‐3^*^	62	CT	TURP	WD	4 (2+2)	0^	
PC12‐7^*^	59	CT	TURP	PD	9 (4+5)	50–80	Y
PC12‐8^*^	73	CT	TURP	—	6 (3+3)	<20	Y
PC22‐576^*^	69	CT	RP	M/PD	7 (3+4)	<20	Y
PC3250‐1^*^	51	CT	RP	PD	9 (4+5)	>80	Y
PC72‐6^*^	62	CT	TURP	W/MD	10 (5+5)	50–80^#^	Y

* Tumor included in our previously published work [4].

^a^ Tumor grade obtained from pathology report;

^b^ Contemporary Gleason score from FFPE tissue block chosen for macro‐dissection; TURP: transurethral resection of the prostate; RP: radical prostatectomy; WD: well differentiated; MD: moderately differentiated; PD: poorly differentiated; –: information was absent in original pathology report; ^proteome removed from differential protein expression analysis due to no cancer present in tissue section; ^^sample failed QC; ^#^two samples were assayed for this individual. Clinical data are representative of the prostate tumor sample with the highest % of malignancy, and the one included in subsequent differential expression analyses.

^c^ Malignant RNA was macro‐dissected, extracted, and assayed separately to benign prostate cells.

We have previously shown that *HOXB13* expression levels do not differ between G84E variant and wildtype tumors [4] and results from this current study confirmed these findings (*p* = 0.09; Figure 1A). The objective of this study was to identify molecular pathways that distinguish HOXB13 G84E variant positive from HOXB13 wildtype tumors. Analysis of the 17,128 quantified genes that passed QC, revealed 799 that were differentially expressed between G84E variant carriers and non‐carriers, as shown in Figure 1B. Of these, 159 were upregulated and 640 downregulated in G84E positive compared with wild‐type tumors. A full list of the differentially expressed genes (DEGs) is provided in Tables S1 and S2.

**FIGURE 1:**
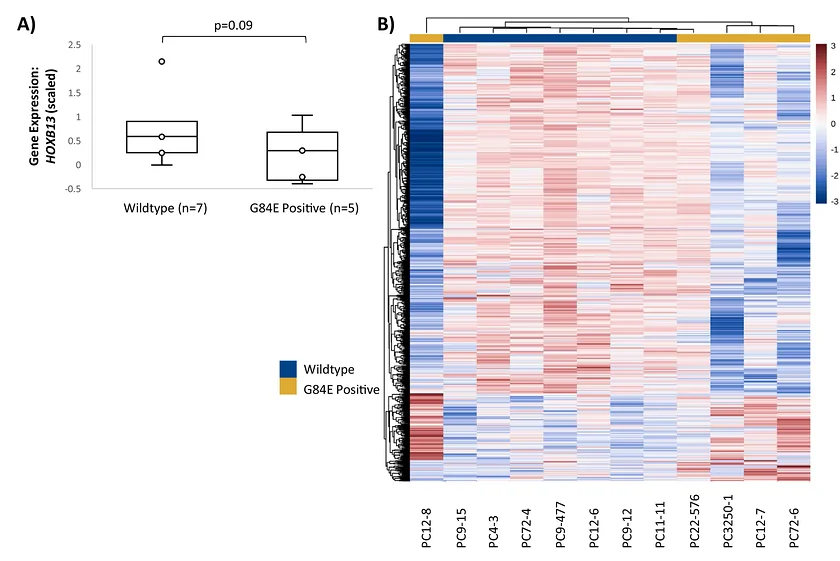
(A) *HOXB13* gene expression (scaled) of G84E‐positive (*n* = 5) and wild‐type tumors (*n* = 7, *p* = 0.09). (B) Heatmap of the 799 genes found to be differentially expressed between G84E‐positive (yellow) and wildtype tumors (blue).

Gene Ontology (GO) analysis of the 799 genes identified five distinct pathways. Consistent with findings reported by Nturubika et al. who demonstrated altered expression of trafficking‐related genes in their analysis of publicly available PrCa RNAseq data [26], we identified the GO pathway, intracellular transport (GO:0046907) as one of t top pathways. Other pathways included protein localization to organelle (GO:0033365), protein transport (GO:0015031), cellular biosynthetic process (GO:0044249), and protein metabolic process (GO:0019538) (Table S3). Notably, enrichment of cellular biosynthetic processes is consistent with the well‐established metabolic rewiring of PrCa cells (as reviewed in Pujana‐Vaquerizo et al. [27]). Further, cluster analysis found that the top cluster of genes (*n* = 297) were involved in nucleic acid metabolic processes and transport, such as RNA and mRNA metabolic processes, translation and RNA splicing (Figure S2 and Table S4). The second cluster included 229 genes as shown in Figure S3, 30 of which mapped to the cellular lipid metabolic process, and these are depicted in the heatmap in Figure 2 (Table S4).

**FIGURE 2:**
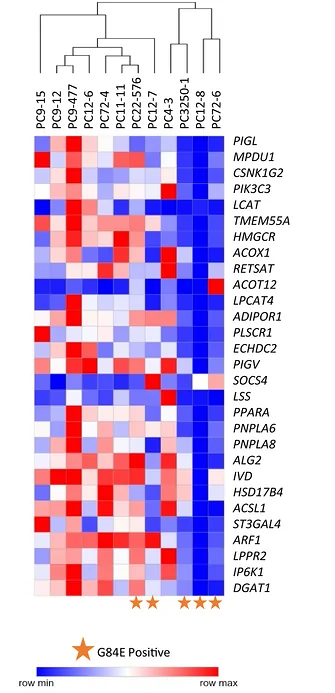
Heatmap of the 30 lipogenesis pathway genes that mapped to the second DEG cluster as shown in Figure S3. Heatmap represents raw gene expression data. HOXB13 G84E‐positive tumors are depicted with a star.

### 2.2 Proteomic Analysis of HOXB13 G84E Variant‐Positive and Wildtype Prostate Tumors

To explore whether there were detectable differences in protein expression between prostate tumors from G84E variant carriers and non‐carriers, data‐independent acquisition mass spectrometry (DIA‐MS) proteomic analysis was performed [28]. A total of 14,908 peptides corresponding to 3180 proteins were quantified. The quantified proteins ranged from approximately 2000 to 3000 proteins per sample. When evaluated, a missing rate of ∼10 to ∼70% per sample was observed, with missingness variable across instruments (Figure S4); however, this effect was reduced after normalization (Figure S5). Proteins with missing data in >50% of the samples were filtered (*n* = 751) and the technical reproducibility of samples was evaluated by the Pearson correlation coefficient (Pearson's *r*) among sample replicates. A high degree of correlation was observed, ranging from 0.83 to 0.91. Of the 3180 proteins detected, 1250 proteins were quantified in >90% of the samples and 140 proteins were quantified in <20% of the samples (Figure S6). In total, 2429 proteins were quantified in 23 prostate tumor samples (Figure S7), including six heterozygous HOXB13 G84E variant‐positive and 17 wildtype tumors (clinical characteristics described in Table 1; PC9‐15 failed QC; PC12‐03 and PC9‐477 were excluded due to no cancer in the tumor block). Uniform manifold approximation and projection (UMAP) analyses revealed that samples clustered based on percentage of cancer in the tissue section (Figure 3A) but did not cluster by HOXB13 G84E carrier status (Figure 3B), Gleason score (≤7 (3+4) vs. ≥7 (4+3); Figure 3C) or age at diagnosis (<65 years vs. ≥65 years; Figure 3D) (other covariates are presented in Figure S8).

**FIGURE 3:**
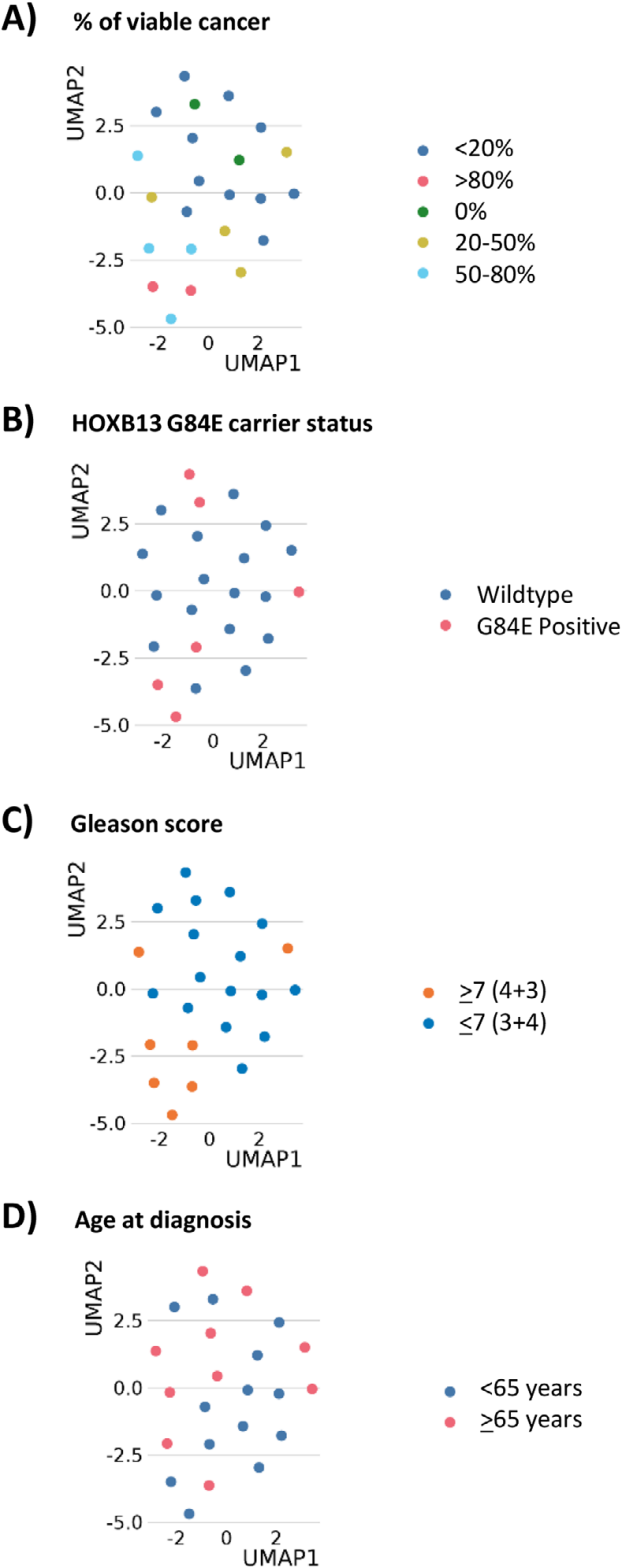
UMAP plots of the filtered protein matrix showing clustering of proteomes based on; (A) percentage (%) of viable cancer, (B) HOXB13 G84E carrier status, (C) Gleason score, and (D) age at diagnosis.

Furthermore, HOXB13 protein expression was on average slightly higher in G84E‐positive tumors (*n* = 5) versus wildtype tumors (*n* = 16), as shown in Figure 4A; however, this was not statistically significant (*p* = 0.23). This is consistent with our previous findings, where we observed no difference in protein levels detected in malignant cells from G84E variant carriers (*n* = 9) and non‐carriers (*n* = 9) by immunohistochemistry [4].

**FIGURE 4:**
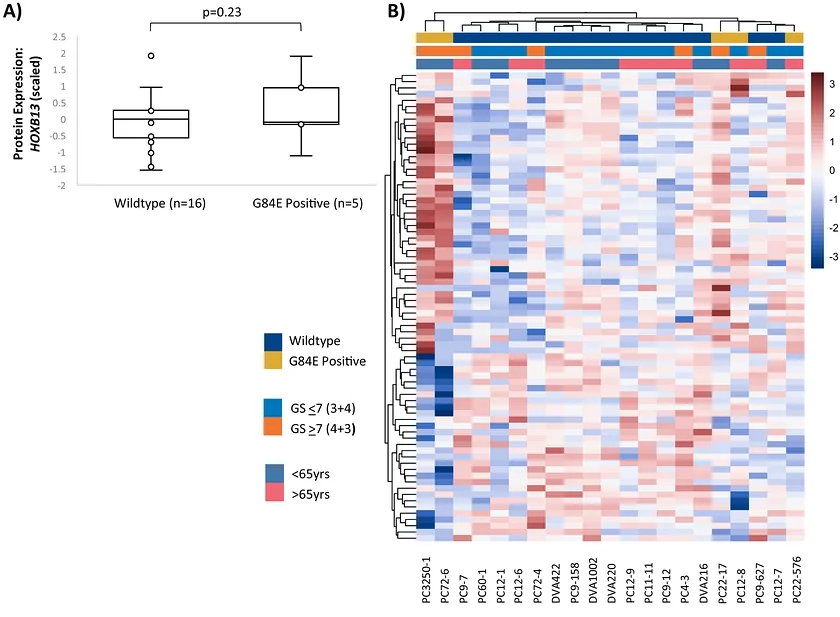
(A) HOXB13 protein expression (scaled) of G84E‐positive (*n* = 5) and wildtype tumors (*n* = 16, *p* = 0.23). (B) Heatmap of the 132 proteins found to be differentially expressed between G84E‐positive and wildtype tumors when percentage of viable cancer was included as a covariate.

Differential protein expression analysis of G84E variant‐positive and wildtype tumors revealed a 232‐protein profile associated with G84E carrier status. A total of 103 proteins were found to be downregulated and 129 were upregulated in the tumors of HOXB13 G84E variant carriers compared with non‐carriers. Analysis was then performed using percentage of cancer as a covariate (as shown in Table 1). This revealed a 132‐protein profile associated with G84E carrier status, including 68 downregulated and 64 upregulated proteins, as shown in the Figure 4B heatmap. As expected, 75 proteins overlapped between these two differential protein expression analyses. A full list of the differentially expressed proteins (DEPs) can be found in Tables S5 and S6.

GO analysis of the 75 overlapping proteins (30 downregulated and 45 upregulated) identified seven main biological processes (Table S7). Consistent with the findings of Lima et al., which examined the proteome of prostate tumors in comparison with normal surrounding tissue [29], we found that of the seven main pathways identified, the purine metabolic pathways (GO:0006144; (GO:0034032; GO:0006163)) and a lipid biosynthesis pathway (GO:0009062) were also prominent. Other pathways included mitochondrial translation elongation (GO:0070125), ribonucleoside bisphosphate metabolic process (GO:0033875), and amide metabolic process (GO:0043603). Cluster analysis revealed that the top cluster consisted of 23 proteins mapping to lipogenesis cellular pathways, including cellular lipid catabolic processes, fatty acid beta‐oxidation and catabolic processes, and short‐chain fatty acid metabolic processes (Table S8). Figure 5A represents the 23 proteins and their relationships. Notably, these processes were upregulated in tumors from G84E variant carriers, see Figure 5B. A second cluster mapped to the desmosome, an intercellular junction complex responsible for adhesion between cells, a key component of which are lipid rafts (Figure 5C and Table S8).

**FIGURE 5:**
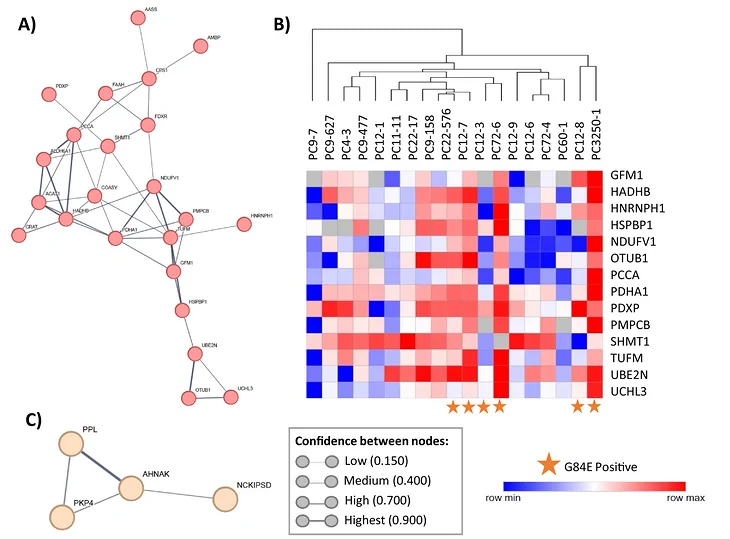
(A) Protein–protein interaction web of DEP cluster one, which included proteins involved in mitochondrial translation, and fatty acid and lipid processes. (B) Heatmap of the 23 proteins in the top DEP cluster (as shown in Figure 5A). Heatmap represents raw protein expression data. HOXB13 G84E‐positive tumors are depicted with a star. (C) Protein–protein interaction web of DEP cluster two which mapped to the desmosome, an intercellular junction that provides strong adhesion between cells.

### 2.3 A Comparison of Transcriptomic and Proteomic Data Identifies Three Genes That are Differentially Expressed According to HOXB13 G84E Carrier Status

To determine whether integration of transcriptome and proteome data could detect a specific G84E‐associated molecular profile, the 799 DEGs and 75 DEPs were compared revealing three overlapping genes: *NCLN, NFIB*, and *VASN*. All were downregulated in G84E variant‐positive tumors at the gene and protein level, except for NCLN, which was downregulated at the gene level and upregulated at the protein level. Heatmaps displayed in Figure 6 show the pattern of expression of these genes (herein “G84E‐like”) across the tumor dataset.

**FIGURE 6:**
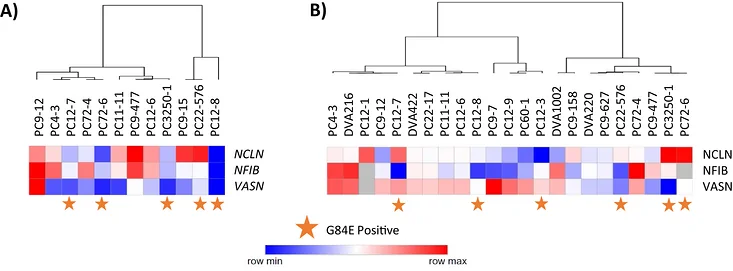
The “G84E‐like” expression signature identified in G84E‐positive tumors consisted of the *NCLN*, *NFIB*, and *VASN* genes. Raw gene expression data are shown in (A) and protein expression data in (B).

Publicly available gene expression data from 493 TCGA tumors were then examined to identify tumors with a similar transcriptomic expression pattern (i.e., concurrent downregulation of *NCLN, NFIB*, and *VASN*). In total, 52 of the 493 TCGA tumors (∼10%) were found to have a “G84E‐like” expression signature. Figure 7A–C display the mRNA expression of *NCLN, NFIB* and *VASN* in the 52 “G84E‐like” signature tumors compared with the remainder of TCGA dataset (*n* = 441). While not statistically significant, the 52 G84E‐like tumors were diagnosed at a slightly younger age (mean of 60 vs. 61 years) compared with the remainder of the dataset (*n* = 441; *p* = 0.17). Similarly, the G84E‐like tumors tended to have lower grade disease (67% had Gleason score ≤7) compared with the remainder of the dataset (58% had Gleason score ≤7).

**FIGURE 7:**
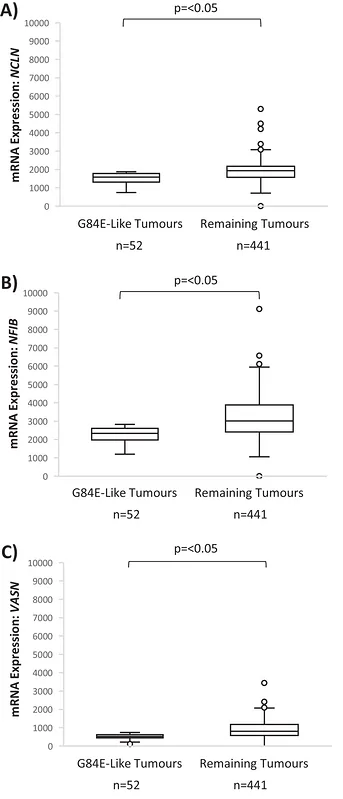
Gene expression of the (A) *NCLN*, (B) *NFIB*, and (C) *VASN* genes in TCGA tumors. In total, 52 tumors were found to have a similar expression pattern to our G84E‐positive tumors (i.e., concurrent downregulation of *NCLN*, *NFIB*, and *VASN*, which are annotated as tumors with a G84E‐like gene signature).

## 3 Discussion

The molecular changes that occur in HOXB13 G84E‐positive tumors have not been examined, with the functionality of the G84E variant only previously examined in vitro. A study by Cardoso and colleagues [30] found no significant differences in tumorigenic characteristics of stably transfected cells overexpressing the G84E protein (compared with cells overexpressing wildtype HOXB13) [30], leaving unanswered questions regarding the role of G84E in tumor development. Computational modeling by Chandrasekaran and colleagues predicted that G84E increases HOXB13 protein stability, leading to an increased half‐life with altered binding patterns and constitutive expression of downstream genes [31]. Here, we used a proteogenomic approach to define the molecular changes occurring in heterozygous HOXB13 G84E variant‐positive tumors. The G84E variant was not associated with detectable differences in HOXB13 expression at the gene and protein levels in prostate tumors, consistent with our previous findings [4]. Previously, we have shown that the variant allele is rarely detectable at the mRNA level in G84E‐positive prostate tumors and when present, is transcribed at lower levels than the wildtype allele [4].

In a novel approach, we sought to map the G84E tumor transcriptome and proteome and compare it with wildtype. A 799‐gene and 75‐protein profile associated with G84E carrier status was identified. Pathway analysis of these data highlighted 202 pathways in the transcriptome data and 57 pathways in the proteome data—the overlapping pathways comprised metabolic and cellular processes, amide biosynthesis and metabolic process, and multiple lipogenesis pathways. This novel finding in patient samples is consistent with recent work by Lu and colleagues [19]. In this study, knockdown of HOXB13 expression in PrCa cell lines resulted in upregulation of lipogenic pathways, as demonstrated by lipid accumulation in cells (detected by oil red staining). This dysregulation was rescued by overexpression of wildtype HOXB13; however, when HOXB13 G84E protein was similarly overexpressed, the phenotype could not be rescued, with sustained expression of lipogenic genes and concomitant lipid accumulation detected [19]. This work demonstrates that the HOXB13 G84E protein is defective in its ability to suppress lipogenic pathways in an in vitro cell model. Here, we provide the first evidence from patient samples that G84E‐positive tumors also show an upregulation of genes in the steroid metabolic, lipid biosynthesis, and fatty acid metabolic pathways.

Integration of transcriptome and proteome data at the individual gene/protein level revealed three overlapping genes, including downregulation of *NCLN*, *NFIB*, and *VASN*, forming a “G84E‐like” expression signature in G84E variant‐positive tumors. Studies in hepatocytes have previously revealed that NFIB knockout is associated with perturbed lipid metabolism [32] and in a mouse model of PrCa, loss of NFIB leads to prostate hyperplasia [33]. Yet, higher NFIB expression drives AR expression in vitro [34] and is associated with metastatic castration resistant PrCa in vivo [35]—indicating the role of this gene is context dependent. VASN (also known as SLITL2) has been implicated in hepatocyte autophagy and regulation of the tightly controlled glycogen/lipid metabolism [36], and the soluble cleaved form of VASN interacts with TGF‐β in endothelial cells impacting signaling in inflammation and in adipocytes, lipogenesis [37]. In one of the few studies evaluating the role of VASN in PrCa, Cui and colleagues found that VASN upregulation promotes epithelial to mesenchymal transition through a key regulator, the YAP/TAZ axis [38]. VASN levels were measured in prostate tumors and serum, with data suggesting that high VASN may have diagnostic value. Here, we found lower VASN expression in G84E‐positive tumors, which tend to exhibit lower‐grade disease, thus supporting the study by Cui et al. [38]. Finally, the *NCLN* gene codes for the Nicalin protein, which together with NOMO, regulates the gamma‐secretase complex [39], a complex associated with lipid rafts and regulated by lipid metabolic pathways. There is currently intense interest in the utility of therapies targeting lipogenesis for the treatment of PrCa. Multiple circulating lipid species have been associated with a PrCa diagnosis and disease progression, including fatty acids and sphingomyelins, amongst others. Testing of a variety of potential therapies targeting cholesterol (statins) and metformin (metabolic syndrome) in clinical trials have produced mixed results; however, there is evidence for efficacy in men with dysregulated lipid metabolism‐associated PrCa [40]. In addition, drugs specifically inhibiting fatty acid synthesis, such as FASN inhibitors, are showing promise as adjuvant therapies [40]. While the results of our study provide further support for the G84E variant causing dysregulation of lipid metabolism, further examination of the molecular pathways is needed. Given the rarity of the HOXB13 G84E variant in the general population, it is expected that there would be very few (if any) G84E carriers in the TCGA dataset. We hypothesized that multiple mechanisms, including somatic mutations, gene amplification, or epigenetic modifications, mimic disruption of the HOXB13 gene afforded by the G84E variant [41]. Our assessment of prostate tumors from TCGA dataset revealed approximately 10% displaying a similar G84E‐like expression signature.

Challenges were encountered in accessing G84E variant‐positive tumors due to the rarity of the variant (frequency of 0.8% in Europeans; gnomAD) and the absence of routine screening of individuals for this variant. Only six HOXB13 G84E‐positive tumors were available for this study; however, insights into rare variant function have been generated previously using a similarly small sample size [42], and in other PrCa proteome studies (reviewed in Sadeesh et al. [21]). While such studies can detect large effect size differences, subtle biological differences may go undetected. Nevertheless, it is important that follow‐up studies are performed in other cohorts of G84E‐positive tumors to evaluate potential biases inherent in our dataset such as possible ascertainment bias, to validate our results and to test generalizability of our findings. It is also noted that few overlapping genes and proteins were detected when comparing the transcriptome and proteome data. This is a recognized phenomenon [43, 44, 45, 46, 47], where prior studies have shown that RNA expression is often a poor predictor of protein expression [48, 49, 50]. In fact, one study suggests that mRNA abundance only explains around 10% of protein abundance [50], which may explain the lack of concordance for the NCLN mRNA and protein levels, and the large difference in the number of DEGs (*n* = 799) compared with DEPs (*n* = 232) identified in our study. These studies highlight the importance of assessing the proteome in addition to inferring gene expression from the transcriptome.

Our study was necessarily conducted in formalin‐fixed, paraffin‐embedded (FFPE) pathology blocks, and while the proteogenomic methodologies were optimized for FFPE and each tissue sample was obtained from the same pathology block; sections selected for RNA and protein analysis were not necessarily adjacent, thus will not be identical in composition due to tumor heterogeneity. PrCa is a multifocal disease and can include heterogeneity within a tumor (reviewed in Haffner et al. [51]). Moreover, proteomic datasets do not represent the full proteome due to technical constraints. This results in fewer DEPs than DEGs, even when considering only protein coding genes, leaving many proteins excluded from analysis.

Notably, the utilization of FFPE in this context allows us to generate data from real world samples, available in the routine pathology setting. While FFPE presents technical challenges due to crosslinks formed between proteins and formaldehyde during sample processing [52], we have shown that our methodology adequately manages this potential limitation. Furthermore, while RNA extracted from FFPE is often degraded, we have an established methodology to successfully generate high‐quality transcriptome data from FFPE samples [53].

Here, we report the first transcriptomes and proteomes generated from heterozygous HOXB13 G84E‐positive prostate tumors and, to the best of our knowledge, this is the first to generate proteome data from familial prostate tumors. Our 799‐gene expression and 75‐protein expression profiles highlighted critical shared pathways, including metabolic and cellular processes, amide biosynthesis, and metabolic process, and significantly, numerous lipogenesis pathways. We also provide evidence from patient samples that G84E‐positive tumors exhibit a perturbed lipogenic proteome profile similar to that identified in an in vitro study by Lu and colleagues [19] and sheds further light on the mechanisms underpinning current interest in the lipid metabolome and PrCa risk. Further investigation of the molecular mechanisms underpinning dysregulation of HOXB13 will provide important insights into the diagnosis of PrCa and the development of novel therapeutic interventions for patients with aberrant HOXB13 function. More broadly addressing perturbed lipogenesis in PrCa should also be a focus of future work. Biomarker profiling of prostate tumors may yield novel diagnostic tools and aide therapy selection for patients with altered lipogenesis profiles.

## 4 Materials and Methods

### 4.1 Study Resources

This study utilized two Tasmanian PrCa cohorts: the *Tasmanian Familial Prostate Cancer Cohort* and the population‐based *Tasmanian Prostate Cancer Case–Control Study* (previously described in Refs. [4, 42, 54]). In total, 26 FFPE prostate tumor samples from 24 PrCa cases were available for this study. Two prostate tumor samples were available for two individuals (PC72‐04 and PC72‐06). The 24 cases included 20 men from the *Familial Cohort* and four from the *Case–Control Study*, comprising six heterozygous *HOXB13* G84E variant carriers (all familial PrCa) and 18 non‐carriers. Sectioned FFPE pathology blocks were histologically reviewed by our team's pathologists (SD and RLB) to provide a contemporary grading of the tumors (SD), mark regions of malignancy (SD and RLB), and estimate the percentage of viable cancer (RLB).

Ethics approval was obtained from the University of Tasmania's Human Research Ethics Committee (H0017040) and written informed consent was obtained from all living participants. For deceased cases, a waiver of consent was obtained to collect prostate tumor samples from pathology laboratories. This study is an observational, comparative case–control analysis of tumor samples from HOXB13 G84E carriers and non‐carriers. Please refer to the STROBE reporting checklist in the Supporting Information.

### 4.2 Nucleic Acid Extraction and Transcriptome Assays

RNA and DNA were co‐extracted from FFPE tissue block sections using the AllPrep DNA/RNA FFPE Kit (Qiagen), with regions of malignant prostate cells macro‐dissected separately from benign cells and prostate stroma. RNA was quantitated using the Agilent TapeStation System and DNA using the Nanodrop ND‐1000 UV–vis spectrophotometer (Nanodrop Technologies). *HOXB13* G84E carrier status was confirmed by Sanger sequencing tumor DNA (forward primer: 5′‐CCGGATAGAAGGCAAACTCA‐3′, reverse primer: 5′‐GCTGATGCCTGCTGTCAACT‐3′; standard MyTaq HS Mastermix (Bioline) thermal cycling conditions with a 62°C annealing temperature). Twelve of the 26 tumor blocks had sufficient RNA from malignant prostate cells for transcriptome analysis.

#### 4.2.1 AmpliSeq^TM^ Transcriptome Assays

RNA was assayed with the AmpliSeq^TM^ Transcriptome Human Gene Expression Kit (ThermoFisher Scientific) and sequenced on the Ion Torrent Proton sequencing system, as previously described [53, 55]. The Ion Torrent Suite v5.02 software was used to map the reads and reads per million values were calculated.

#### 4.2.2 Data Pre‐Processing for Differential Gene Expression Analysis

Gene expression values of the 20,810 genes captured by the AmpliSeq^TM^ Kit were log2 transformed and standardized to the *z*‐score (Figure S1). In short, *z*‐score normalization was performed across all genes so that relative expression levels of each gene could be assessed between all samples. There were 860 genes in the dataset that returned either no detectable expression in all samples or had values in only a single sample; hence, these genes were removed from the data matrix. Furthermore, genes with missing data in >50% of the samples were removed, leaving 17,128 genes for differential gene expression analysis.

### 4.3 Protein Extraction and Mass Spectrometry

Microtome sections from 26 prostate tumor samples were prepared as previously described [28]. All samples were processed at the same time to avoid batch effects, and the data were acquired on two mass spectrometers. HEK293 digestion controls were included. In this dataset, quality checks using UMAP and multidimensional scaling plots and replicate correlation were performed to evaluate for any batch effects. The instrument‐related batch effect observed in Figure S5A was resolved after data normalization using DIA‐NN (RT‐based), as described below. No further batch correction was required post DIA‐NN normalization. Furthermore, an Eksigent nanoLC 425 HPLC (Sciex) was coupled to a 6600 Triple TOF (Sciex) mass spectrometer. The DIA‐MS details were the same as described by Lucas et al. [28]. See Supporting Information for variable windows utilized. Mass spectrometry runs were randomized and run in technical duplicates. Each sample was spiked with unlabeled, non‐human peptides as retention time (RT) standards.

#### 4.3.1 Spectral Library Generation and SWATH Data Extraction

Mass spectrometry data were searched using DIA‐NN 1.8 [56]. Canonical SWISS‐PROT entries were extracted from Uniprot for *Homo sapiens*. The spectral reference library (SRL) was constructed using all SWATH runs and the *Homo sapiens* FASTA file with DIA‐NN. The final SRL, used for searching the data, contained 4547 proteins and 21,422 peptides.

For SWATH data extraction, DIA‐NN was employed using RT‐dependent normalization and data were extracted for 26 samples from 24 PrCa cases. Samples were run in technical duplicates with a total of 50 SWATH runs (two samples were only run once). In addition to patient samples, six HEK293 control runs were also added to the dataset. Therefore, a total of 56 SWATH runs were searched using the SRL.

#### 4.3.2 Data Pre‐Processing for Differential Protein Expression Analysis

From DIA‐NN search results, the precursor data were filtered for proteotypic peptides only with Global.Q.Value < 0.01. Proteins were then quantified using maxLFQ, with default parameters [57] and implemented using the DIA‐NN R Package (https://github.com/vdemichev/diann‐rpackage). Prior to downstream data analysis, data were log2 transformed, and sample replicates were merged to generate the final protein matrix. Where two prostate tumor samples were available from one individual (PC72‐04 and PC72‐06), the sample with the highest percentage of viable cancer was included in subsequent analyses.

### 4.4 Differential Expression Analysis

Gene and protein expression data were standardized to *z*‐scores. To determine differences in expression between HOXB13 G84E variant‐positive and wildtype tumors, the Empirical Bayes moderated *t*‐statistic in the *limma* R package (version 3.54.1) was used to calculate *p* values. Limma assumes a *t*‐distribution for the test statistic, and the empirical Bayes moderation makes the estimate more robust. Significant DEGs and DEPs were selected using a raw *p* value < 0.05 and a log fold change (expressed as the difference in the group means) cut‐off of ±0.5. Volcano plots were generated using the R package, *ggplot2* (version 3.4.1) and heatmaps were generated using the R package, *pheatmap* (version 1.0.12). For heatmaps, the complete linkage clustering algorithm was used, along with Euclidean distance as the distance measure. Cluster‐associated heatmaps (such as Figures 2, 5B, and 6) were generated using Morpheus, https://software.broadinstitute.org/morpheus.

### 4.5 Functional Enrichment Analysis

GO enrichment analysis of the DEGs and DEPs was performed using the PANTHER19.0 Classification System [58, 59] available through Gene Ontology Unifying Biology (https://geneontology.org/docs/go‐enrichment‐analysis/, release November 3, 2024) [60, 61]. Cluster analysis to predict protein–protein interactions was performed using STRING (https://string‐db.org/, version 11.5). The type of interactions between proteins was indicated by the network connection line, as determined by curated databases or experimentally. The input parameters were *p* value <0.05, minimum gene count of 3, and an interaction score of 0.4 (and an enrichment score >1 for the protein data). kmeans clustering was used to identify the top two clusters from each analysis.

### 4.6 TCGA Dataset

The TCGA dataset utilized in this study consisted of somatic data from 493 radical prostatectomy prostate adenocarcinoma samples. The data were accessed through the Pan‐Cancer Atlas (PanCanAtlas) initiative [62, 63], as previously described [55]. Here, mRNA expression profiles (batch normalized from Illumina HiSeq_RNASeqV2) of *NCLN, NFIB*, and *VASN* were sourced through the cBioPortal for Cancer Genomics (https://www.cbioportal.org/datasets). Here, the median expression value of each gene was determined, and tumors with reduced expression of *NCLN, NFIB*, and *VASN* (in comparison with the median) were identified. These tumors were deemed to have a “G84E‐like” expression signature given their similar gene expression profile to G84E‐positive tumors. Clinicopathological features of disease, including Gleason score and age at diagnosiswere statistically assessed between the tumors with the G84E‐like expression signature and the remainder of the dataset. *p* Values <0.05 were considered statistically significant.

## Author Contributions

KR and JRM contributed to sample preparation for both analyses. SW and PGH contributed to sample preparation and experiment for proteome analysis. KR and CJ contributed to the transcriptome data analysis, and ZN, ATA, PJR, and QZ contributed to the proteome data analysis. SD and RLB reviewed the tumor samples. KR wrote the original draft and ZN, ATA, PGH, QZ, LMF, and JLD contributed to the manuscript. LMF and JLD conceived the study, and KR, RRR, and JLD led the study. All co‐authors approved the final manuscript.

## Funding

KR waspreviously supported by a Cancer Council Tasmania/Evelyn Pedersen Fellowship, a Cancer Council Tasmania Joy & Robert Coghlan/College of Health and Medicine Postdoctoral Research Fellowship, an Australian Government Research Training Program Scholarship and a Cancer Council Tasmania Evelyn Pedersen Elite Research Scholarship. JLD is supported by a Select Foundation Cancer Research Fellowship and was previously supported by an Australian Research Council Future Fellowship. LMF is supported by Sir Harold Cuthbertson Foundation and Gerald Harvey Principal Research Fellowships and was previously supported by a Cancer Council Tasmania/College of Health and Medicine Senior Research Fellowship.

The Tasmanian Familial Prostate Cancer Study has been supported by the Tour de Cure, Royal Hobart Hospital Research Foundation, Cancer Council Tasmania, Cancer Australia, The Mazda Foundation, Perpetual Trustees, Max Bruce Trust, The Estate of Dr RA Parker, the Tasmanian Community Fund, and the Robert Malcolm Familial Prostate Cancer Bequest.

The work at ProCan was supported by the Australian Cancer Research Foundation, Cancer Institute New South Wales (NSW) (2017/TPG001, REG171150), NSW Ministry of Health (CMP‐01), The University of Sydney, Cancer Council NSW (IG 18‐01), Ian Potter Foundation, an NHMRC Fellowship to PJR (GNT1137064), and the Medical Research Futures Fund (MRFF‐PD), and was done under the auspices of a Memorandum of Understanding between the Children's Medical Research Institute and the U.S. National Cancer Institute's International Cancer Proteo‐genomics Consortium, which encourages cooperation among institutions and nations in proteogenomic cancer research in which datasets are made available to the public. The aforementioned funders had no involvement in the study design, data collection, analysis, interpretation of data, manuscript preparation, or the decision to publish.

## Ethics Statement

This study was ethically approved by the University of Tasmania's Human Research Ethics Committee (H0017040) and written informed consent was obtained from all participating individuals. For deceased cases, a waiver of consent was obtained to collected prostate tumor samples from pathology laboratories.

## Conflicts of Interest

SD is employed by Sonic Healthcare. RLB has received research support from Illumina for work unrelated to this study. The other authors declare no conflicts of interest.

## Supporting information




**Supporting File 1**: mco271078‐sup‐0001‐SuppMat.docx

**Supporting File 2**: mco271078‐sup‐0002‐Tables.xlsx

## Data Availability

Additional information can be found in the Supporting Information. The mass spectrometry proteomics data have been deposited to the ProteomeXchange Consortium via the PRIDE [64] partner repository with the dataset identifier PXD051471.
