# Supplementary material for: Applying Proteogenomics to Characterize Molecular Features Associated With a High‐Risk HOXB13 Gene Variant in Prostate Cancer Tumors
Source: MedComm (2020). 2026 Oct 21;7(11):e71078. doi: 10.1002/mco2.71078 (PMC13687195; doi:10.1002/mco2.71078)
Supplement: Supplementary file 1 [file MCO2-7-e71078-s002.docx]

**Applying proteogenomics to characterise molecular features associated with a high-risk HOXB13 gene variant in prostate cancer tumours**

Kelsie Raspin^1^, Zainab Noor^2^, Adel T Aref^2^, James R Marthick^1^, Chol-hee Jung^3^, Shaun Donovan^4^, Steve Williams^2^, Peter G Hains^2^, Phillip J Robinson^2^, Rosemary L Balleine^2^, Qing Zhong^2^, Roger R Reddel^2^, Liesel M FitzGerald^1^ & Joanne L Dickinson^1,*^

^1^Menzies Institute for Medical Research, University of Tasmania, Hobart, Tasmania, Australia

^2^ProCan®, Children's Medical Research Institute, Faculty of Medicine and Health, The University of Sydney, Westmead, New South Wales, Australia

^3^Melbourne Bioinformatics, The University of Melbourne, Parkville, Victoria, Australia

^4^Sonic Healthcare, Hobart, Tasmania, Australia

* To whom correspondence should be addressed. Tel: +61 3 6226 7622; Email: [jo.dickinson@utas.edu.au](mailto:jo.dickinson@utas.edu.au)

**SUPPLEMENTARY FIGURES**

**A)**

**B)**

**Density**

**Quantified Genes**

**Samples**

**Logged Scaled Intensity**

**Supplementary Figure 1.** Intensity distribution of the AmpliSeq data (**A**) and genes quantified in fraction of samples (**B**). Supplementary Figure 1B shows that ~13,000 genes (from the total number of quantified genes) were quantified in >90% of samples. Thus, a large proportion of total quantifiable genes were available for analysis in a large set of samples.

**Confidence between nodes:**

Low (0.150)

Medium (0.400)

High (0.700)

Highest (0.900)

**Supplementary Figure 2.** Protein-protein interaction web of DEG cluster one, which included genes involved in nucleic acid metabolic processes and transport, such as RNA and mRNA metabolic processes, translation and RNA splicing.

**Confidence between nodes:**

Low (0.150)

Medium (0.400)

High (0.700)

Highest (0.900)

**Supplementary Figure 3.** Protein-protein interaction web of DEG cluster two which mapped to the cellular lipid metabolic proces

**Supplementary Figure 4.** Protein/peptide count in the non-normalized peptide matrix per sample. PC9-15 had poor overall data quality, with >70% missingness at the protein level.

**A)**

**B)**

**C)**

**Supplementary Figure 5.** Missing rate per sample. (**A**) Normalised peptide matrix. (**B**) Normalised protein matrix all replicates. (**C**) Normalised protein matrix merged sample replicates.

**Missing Rate**

**Density**

**Supplementary Figure 6.** Missing rate per individual on each machine (M1, M2) in the non-normalized peptide matrix.

**B)**

**A)**

**Coordinate 2**

**Coordinate 2**

0

-100

-200

-100	0

-200

-100	0

**Coordinate 1**

**Coordinate 1**

**Supplementary Figure 7.** MDS plot of non-normalised (**A**) and normalised peptides (**B**).

**A)**

**B)**

**Density**

**Quantified Proteins**

**Samples**

**Logged Scaled Intensity**

**Supplementary Figure 8.** Standardised matrix intensity distribution of the proteome data (**A**) and proteins quantified in fraction of samples (**B**).

**A)**

**B)**

**Density**

**Quantified Proteins**

**Samples**

**Logged Scaled Intensity**

**Supplementary Figure 9.** Standardised matrix intensity distribution of the DIA-NN protein data (**A**) and proteins quantified in fraction of samples (**B**).

**Supplementary Figure 10.** UMAP plots of the filtered protein matrix showing clustering of proteomes based on percentage, (**A**) malignant/benign regions in tumour section, (**B**) % of necrosis, (**C**) assayed section consistent with diagnosis, and (**D**) lymphocytic infiltrate.

**SUPPLEMENTARY INFORMATION**

**Variable window information for SWATH mass spectrometry:**

Start m/z,	End m/z	Collision Energy Spread (CES)

399.5	406.5	5

405.5	412.5	5

411.5	418.5	5

417.5	424.5	5

423.5	430.5	5

429.5	436.5	5

435.5	442.5	5

441.5	448.5	5

447.5	454.5	5

453.5	459.5	5

458.5	464.5	5

463.5	469.5	5

468.5	474.5	5

473.5	479.5	5

478.5	484.5	5

483.5	489.5	5

488.5	494.5	5

493.5	499.5	5

498.5	504.5	5

503.5	509.5	5

508.5	514.5	5

513.5	519.5	5

518.5	524.5	5

523.5	529.5	5

528.5	534.5	5

533.5	539.5	5

538.5	544.5	5

543.5	549.5	5

548.5	554.5	5

553.5	559.5	5

558.5	564.5	5

563.5	569.5	5

568.5	574.5	5

573.5	579.5	5

578.5	584.5	5

583.5	589.5	5

588.5	594.5	5

593.5	599.5	5

598.5	604.5	5

603.5	609.5	5

608.5	614.5	5

613.5	619.5	5

618.5	624.5	5

623.5	629.5	5

628.5	634.5	5

633.5	639.5	5

638.5	644.5	5

643.5	649.5	5

648.5	654.5	5

653.5	660.5	5

659.5	666.5	5

665.5	672.5	5

671.5	678.5	5

677.5	684.5	5

683.5	690.5	5

689.5	696.5	5

695.5	702.5	5

701.5	708.5	5

707.5	714.5	5

713.5	720.5	5

719.5	726.5	5

725.5	732.5	5

731.5	738.5	5

737.5	744.5	5

743.5	750.5	5

749.5	756.5	5

755.5	763.5	5

762.5	770.5	5

769.5	777.5	5

776.5	784.5	5

783.5	791.5	5

790.5	798.5	5

797.5	805.5	5

804.5	812.5	8

811.5	819.5	8

818.5	826.5	8

825.5	834.5	8

833.5	842.5	8

841.5	850.5	8

849.5	858.5	8

857.5	867.5	8

866.5	876.5	8

875.5	885.5	8

884.5	894.5	8

893.5	903.5	8

902.5	914.5	8

913.5	925.5	8

924.5	936.5	8

935.5	950.5	8

949.5	964.5	8

963.5	978.5	8

977.5	992.5	8

991.5	1011.5	10

1010.5	1030.5	10

1029.5	1054.5	10

1053.5	1078.5	10

1077.5	1117.5	10

1116.5	1156.5	10

1155.5	1200.5	10

1199.5	1249.5	10

**References**

1.	Tang Z, Li C, Kang B, et al. GEPIA: a web server for cancer and normal gene expression profiling and interactive analyses. *Nucleic Acids Res*. 2017;45:W98-W102.
